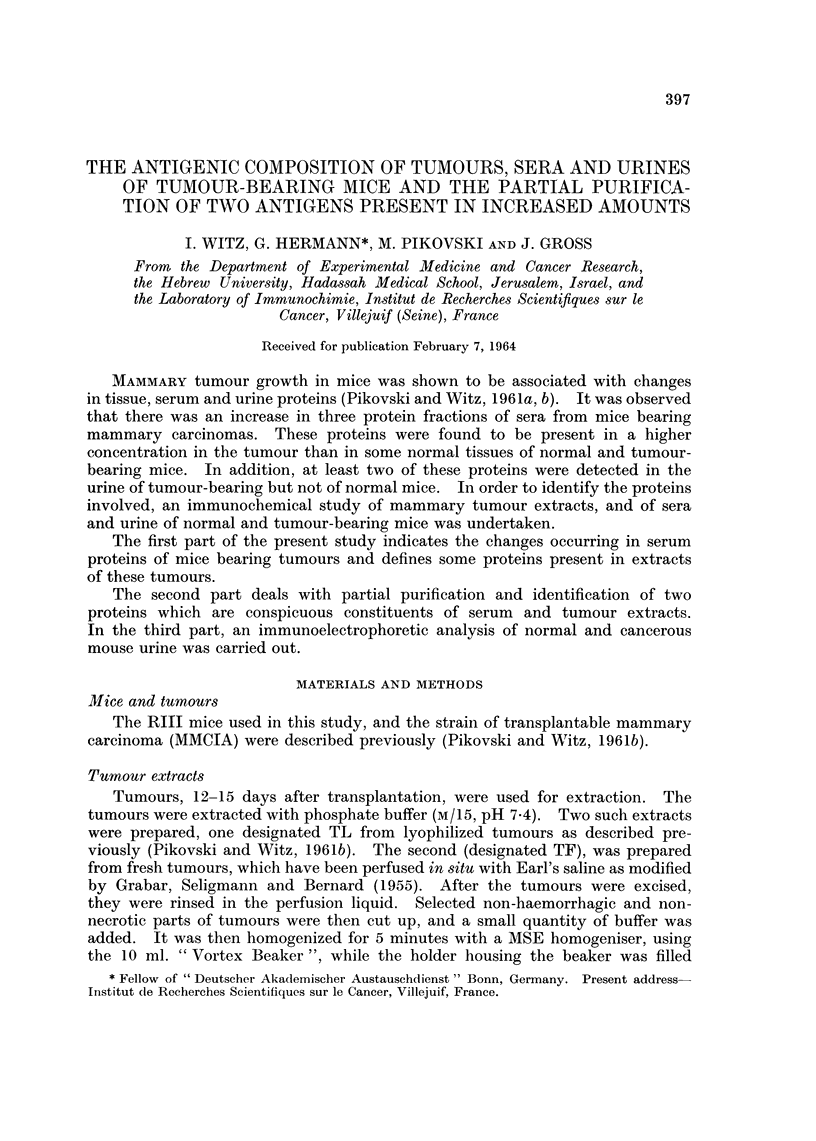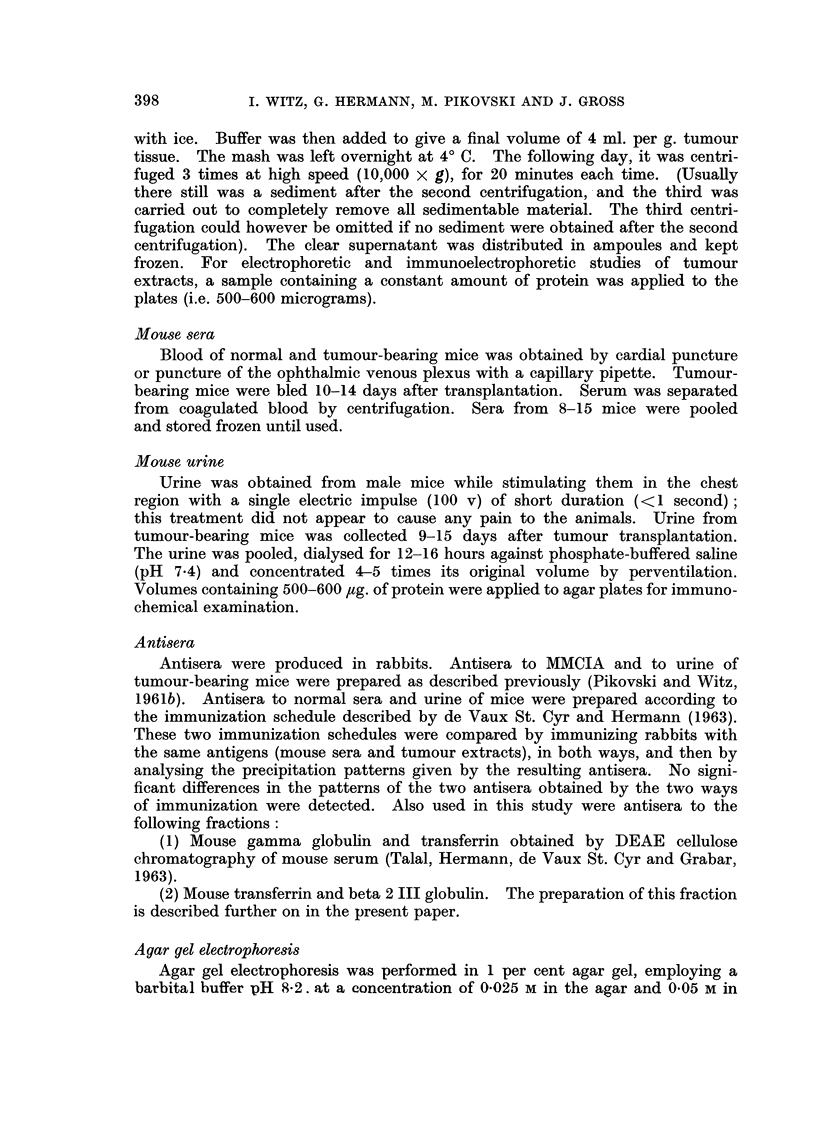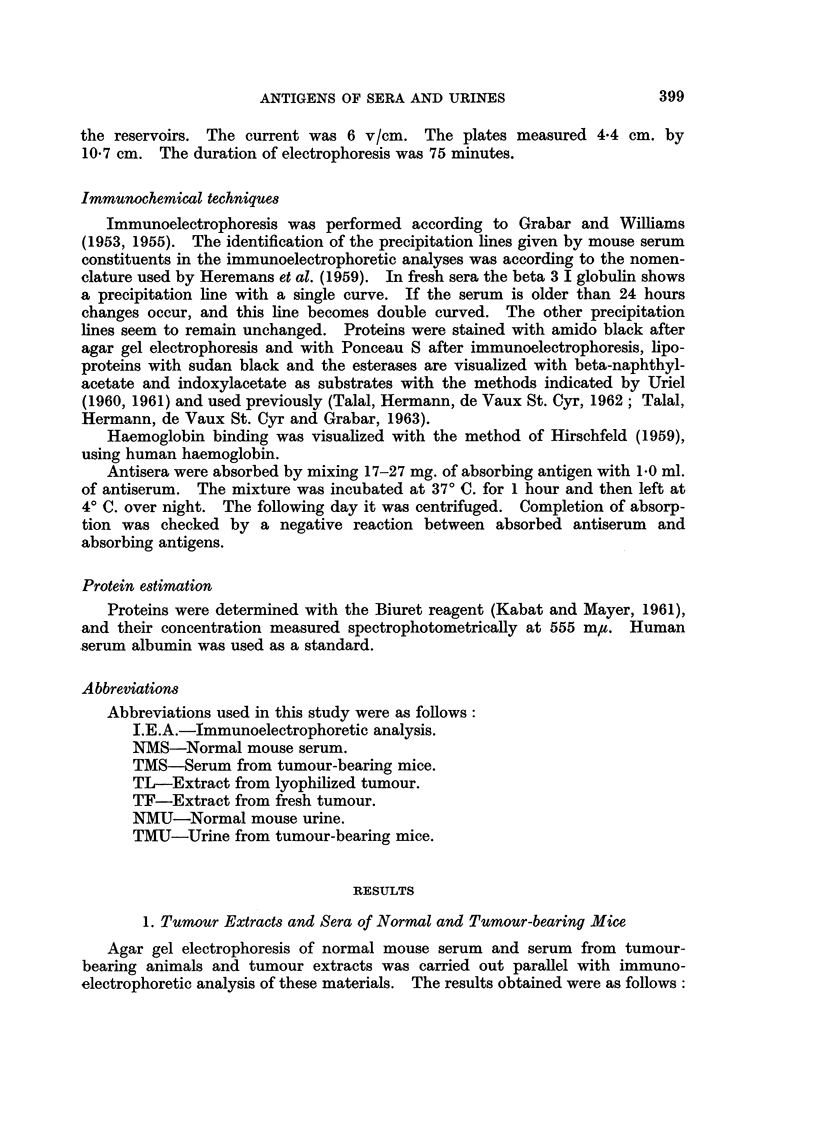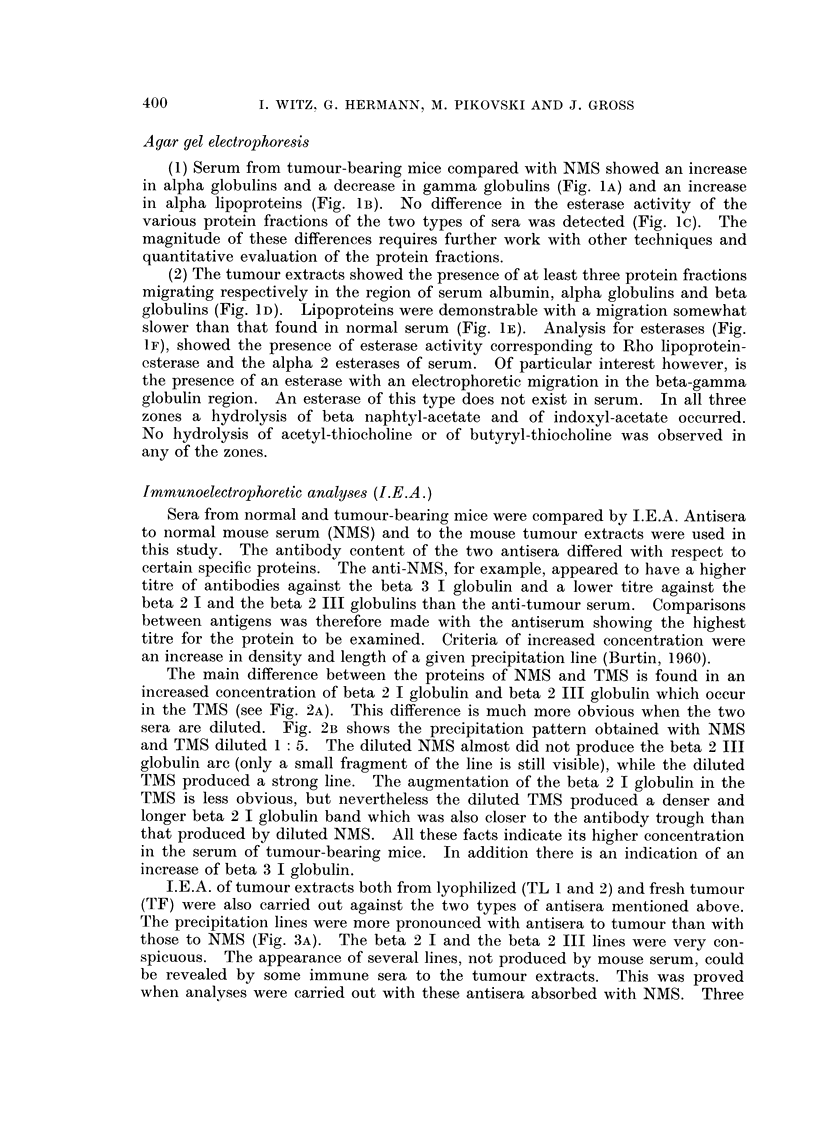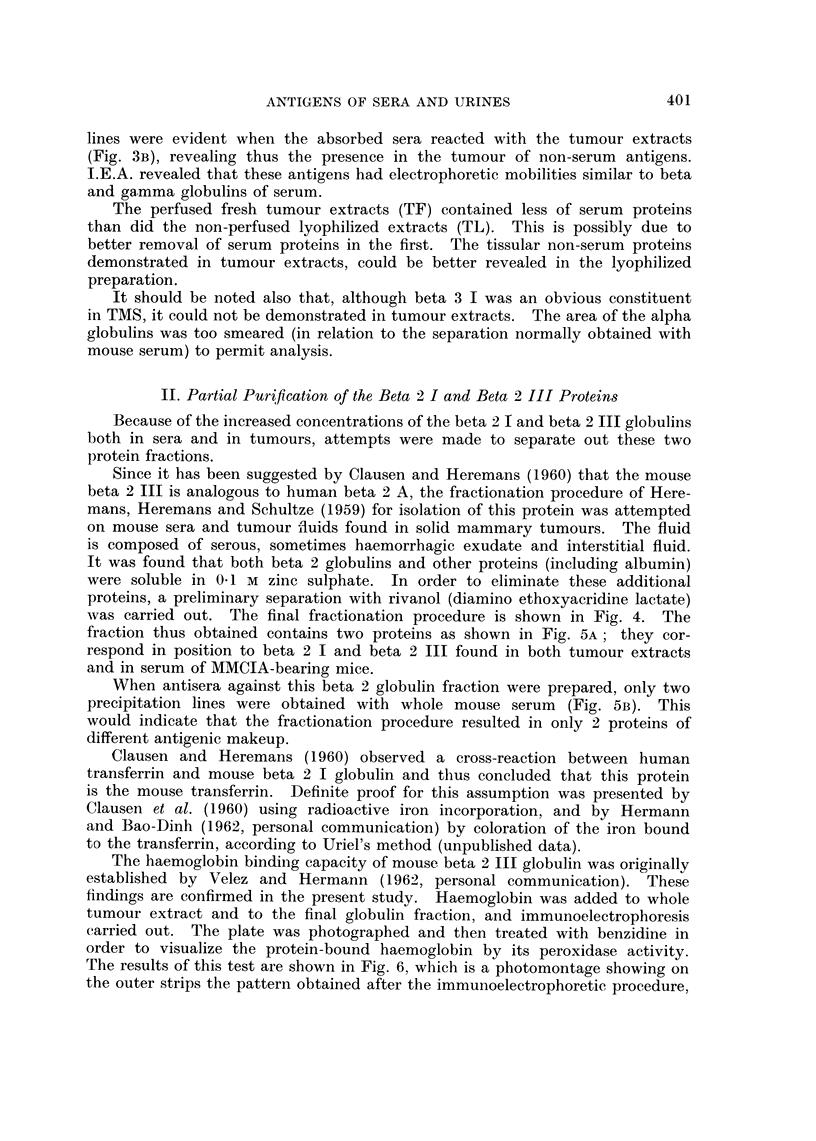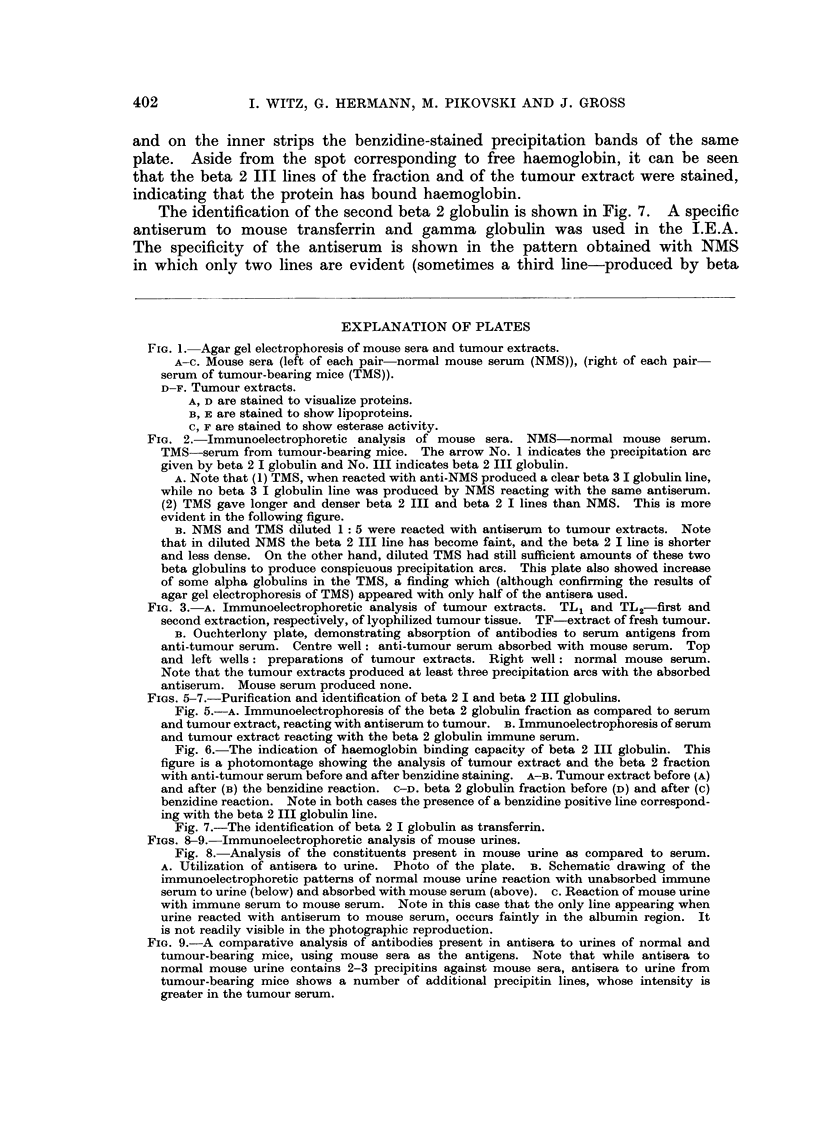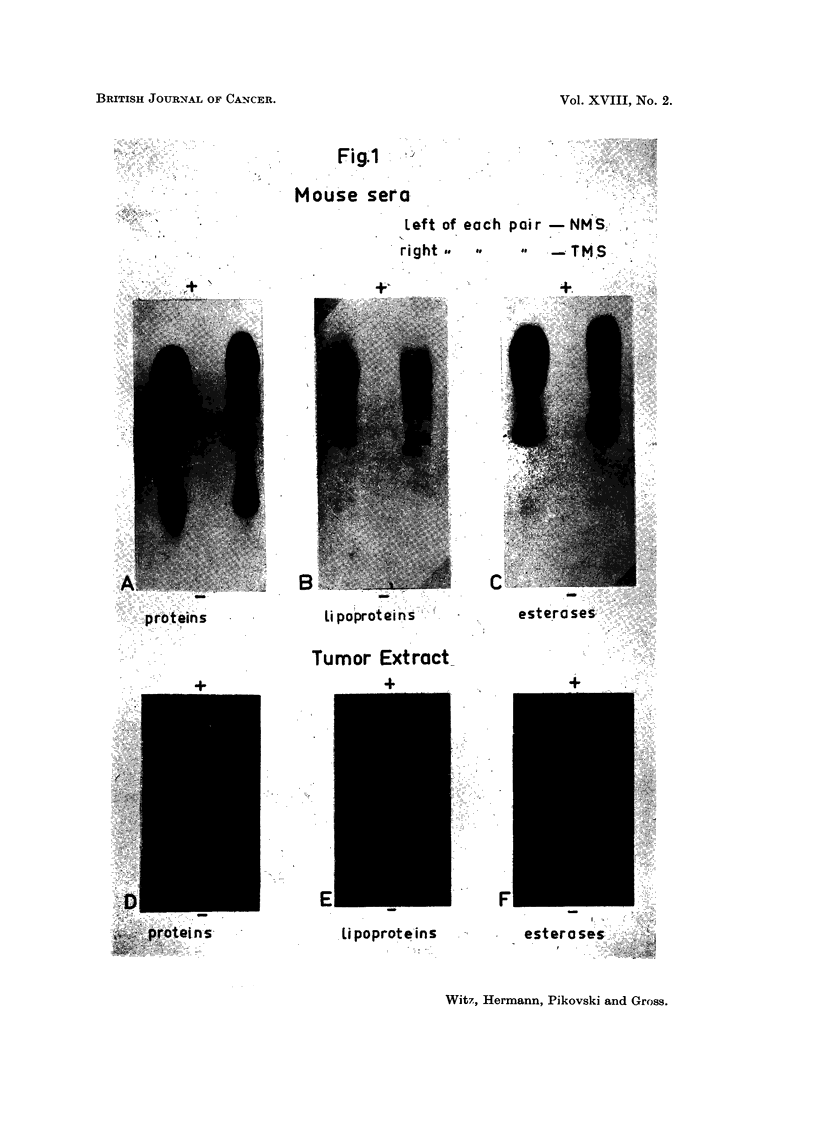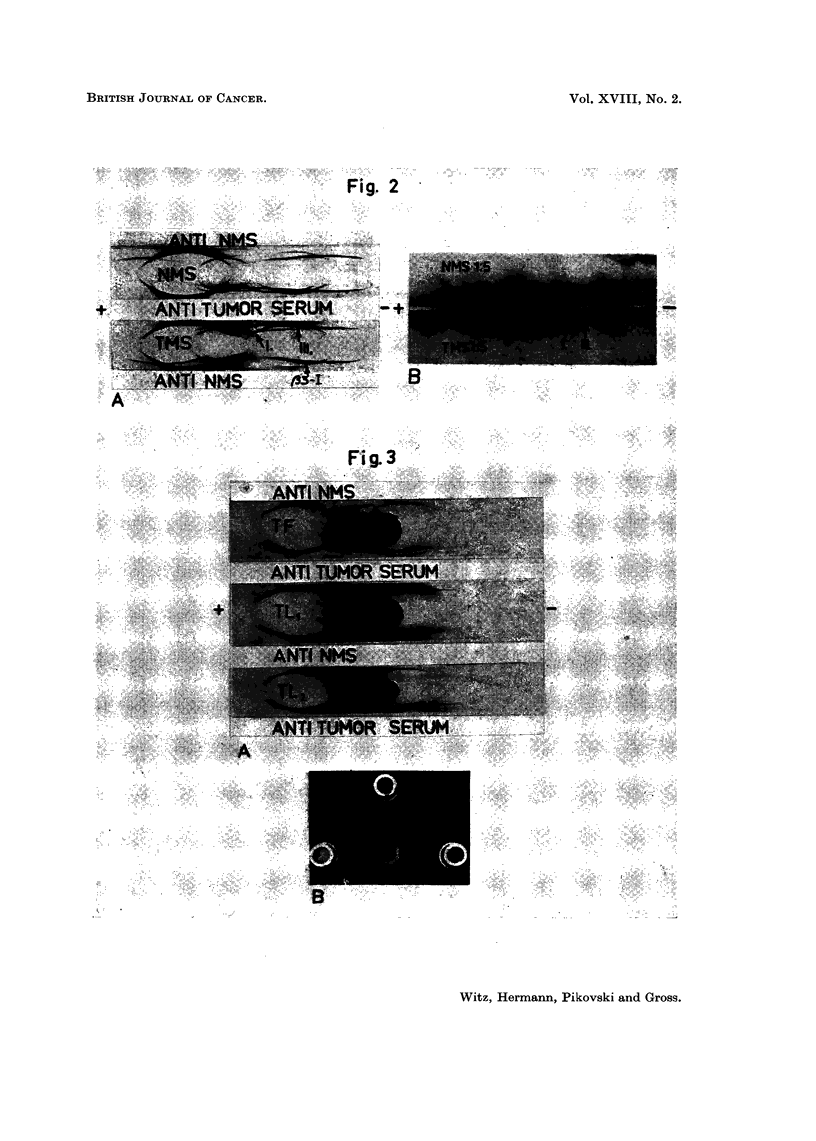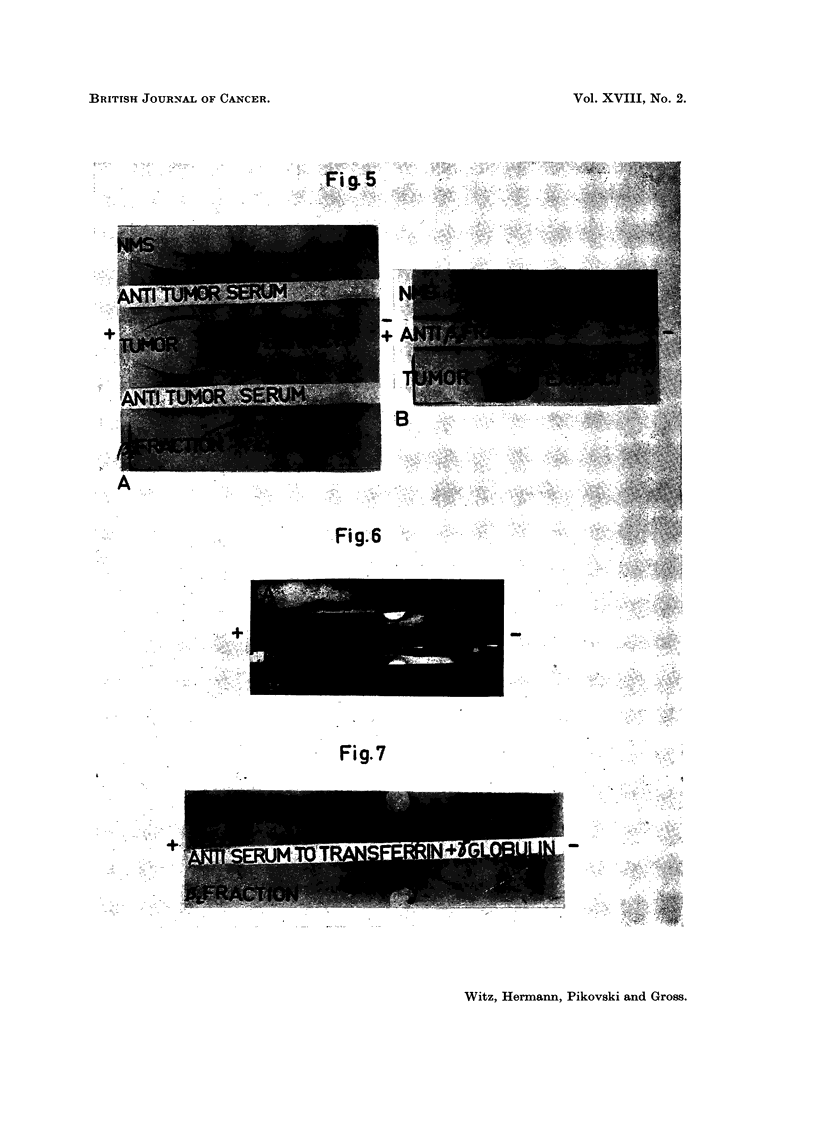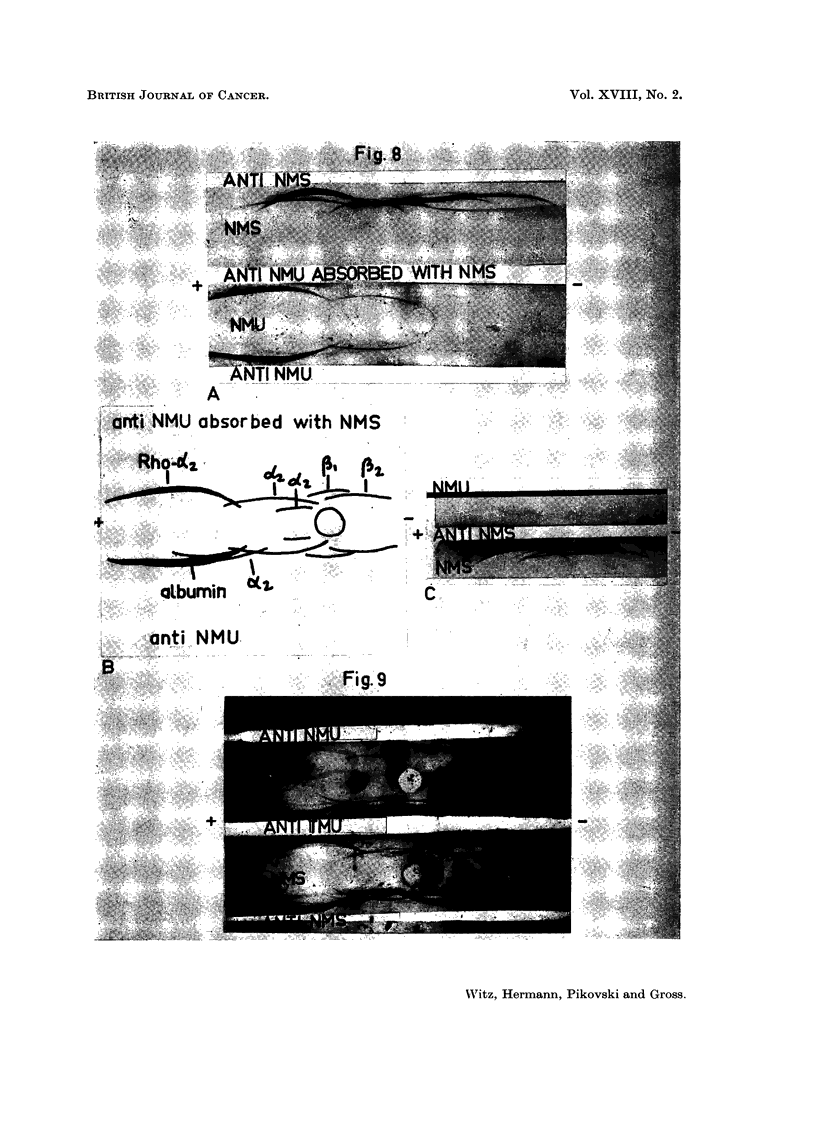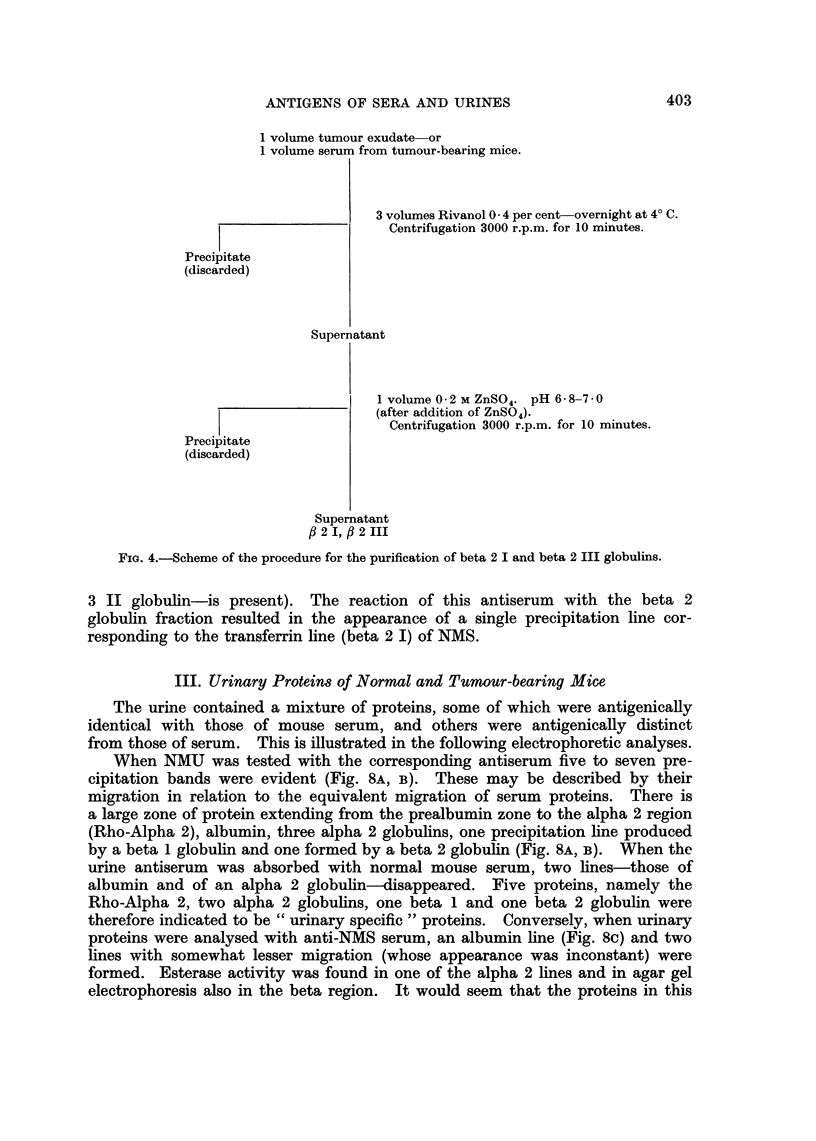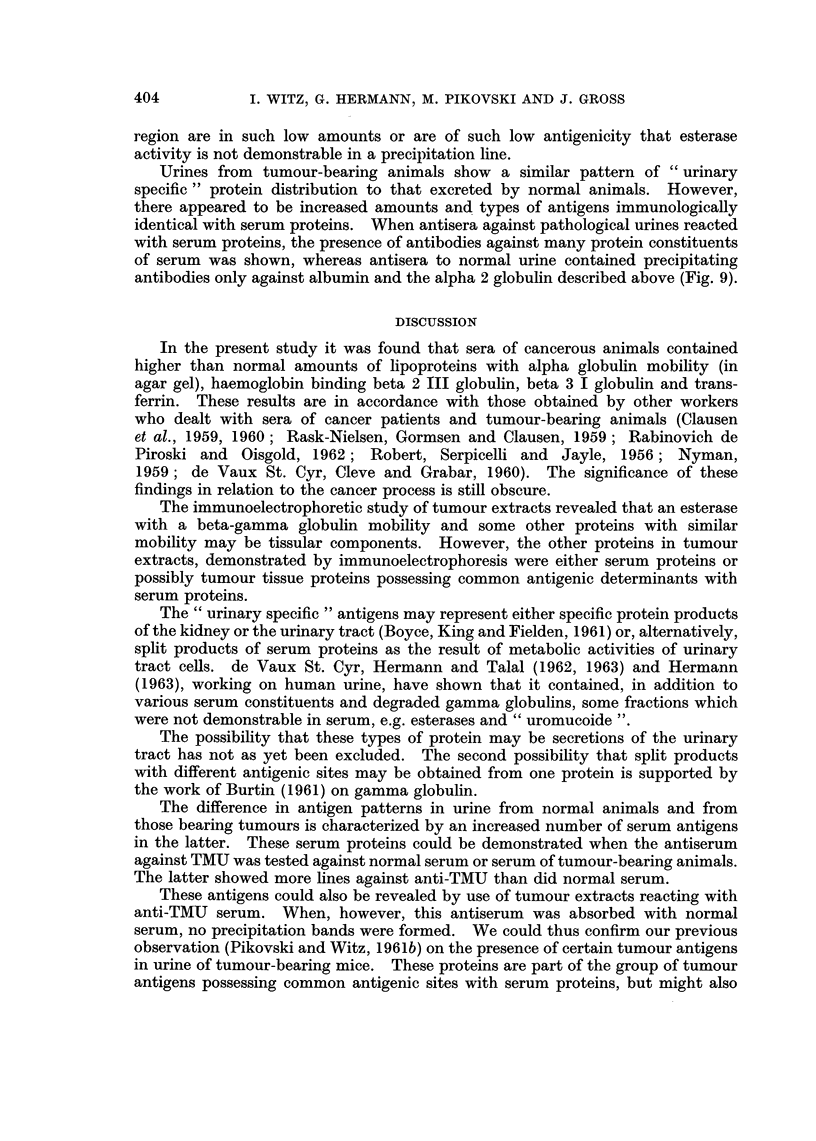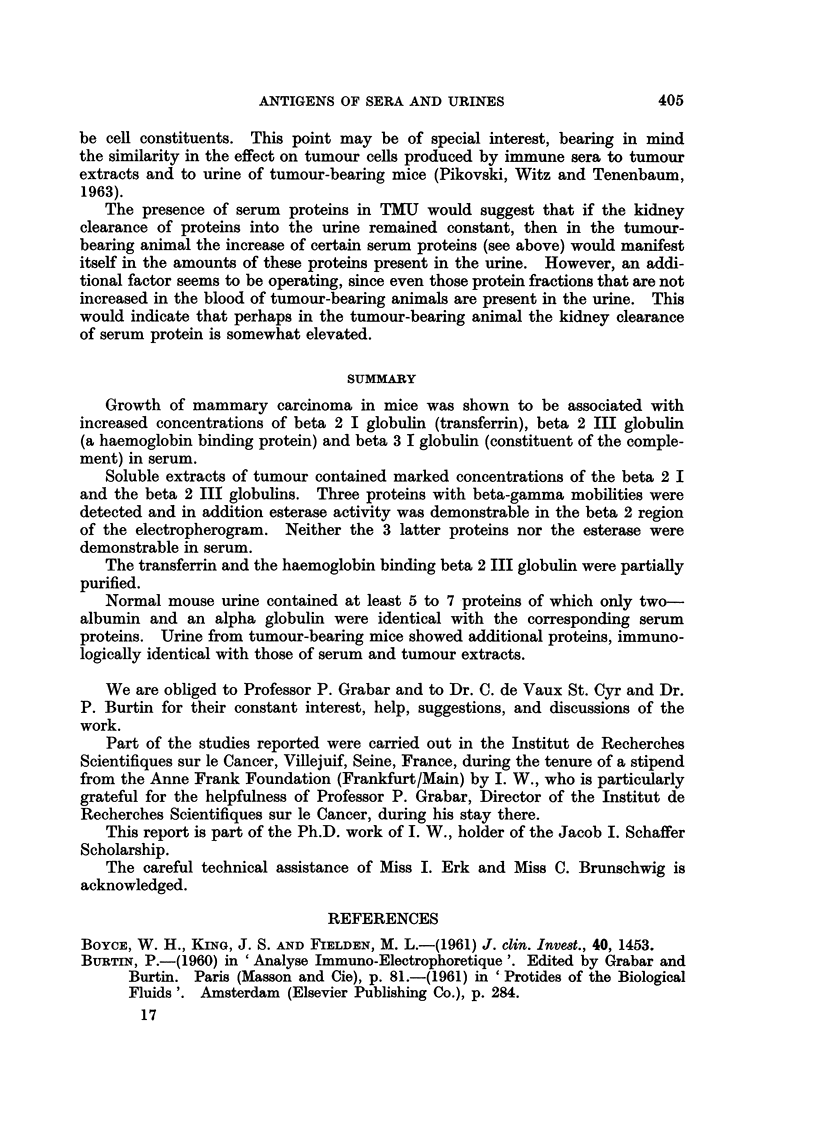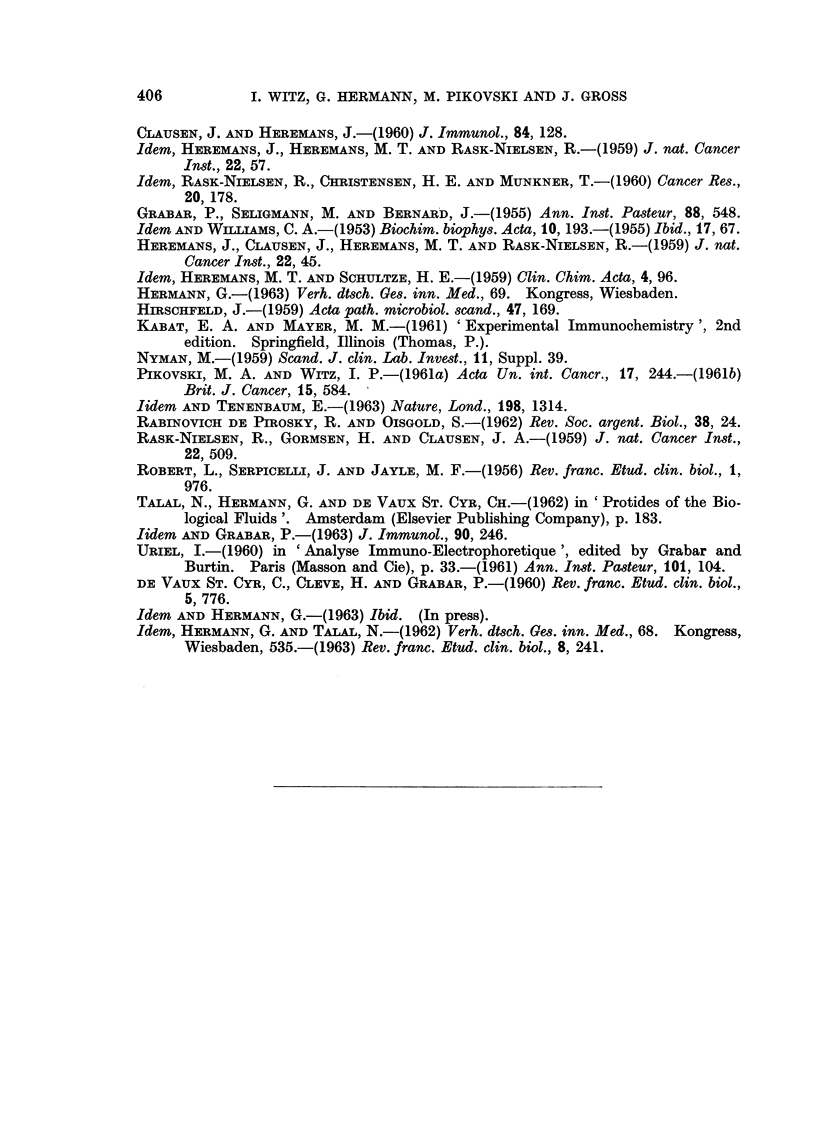# The Antigenic Composition of Tumours, Sera and Urines of Tumour-Bearing Mice and the Partial Purification of Two Antigens Present in Increased Amounts

**DOI:** 10.1038/bjc.1964.46

**Published:** 1964-06

**Authors:** I. Witz, G. Hermann, M. Pikovski, J. Gross

## Abstract

**Images:**


### THE ANTIGENIC COMPOSITION OF TUMOURS, SERA AND URINES OF TUMOUR-BEARING MICE AND THE PARTIAL PURIFICATION OF TWO ANTIGENS PRESENT IN INCREASED AMOUNTS

I. WITZ, G. HERMANN*, M. PIKOVSKI AND J. GROSS

*From the Department of Experimental Medicine and Cancer Research, the Hebrew University, Hadassah Medical School, Jerusalem, Israel, and the Laboratory of Immunochimie, Institut de Recherches Scientifiques sur le Cancer, Villejuif (Seine), France*

Received for publication February 7, 1964

MAMMARY tumour growth in mice was shown to be associated with changes in tissue, serum and urine proteins (Pikovski and Witz, 1961*a*, *b*). It was observed that there was an increase in three protein fractions of sera from mice bearing mammary carcinomas. These proteins were found to be present in a higher concentration in the tumour than in some normal tissues of normal and tumour-bearing mice. In addition, at least two of these proteins were detected in the urine of tumour-bearing but not of normal mice. In order to identify the proteins involved, an immunochemical study of mammary tumour extracts, and of sera and urine of normal and tumour-bearing mice was undertaken.

The first part of the present study indicates the changes occurring in serum proteins of mice bearing tumours and defines some proteins present in extracts of these tumours.

The second part deals with partial purification and identification of two proteins which are conspicuous constituents of serum and tumour extracts. In the third part, an immunoelectrophoretic analysis of normal and cancerous mouse urine was carried out.

### MATERIALS AND METHODS

### *Mice and tumours*

The RIII mice used in this study, and the strain of transplantable mammary carcinoma (MMCIA) were described previously (Pikovski and Witz, 1961*b*).

### *Tumour extracts*

Tumours, 12–15 days after transplantation, were used for extraction. The tumours were extracted with phosphate buffer (M/15, pH 7·4). Two such extracts were prepared, one designated TL from lyophilized tumours as described previously (Pikovski and Witz, 1961*b*). The second (designated TF), was prepared from fresh tumours, which have been perfused *in situ* with Earl's saline as modified by Grabar, Seligmann and Bernard (1955). After the tumours were excised, they were rinsed in the perfusion liquid. Selected non-haemorrhagic and non-necrotic parts of tumours were then cut up, and a small quantity of buffer was added. It was then homogenized for 5 minutes with a MSE homogeniser, using the 10 ml. " Vortex Beaker ", while the holder housing the beaker was filled

* Fellow of " Deutscher Akademischer Austauschdienst " Bonn, Germany. Present address—Institut de Recherches Scientifiques sur le Cancer, Villejuif, France.

with ice. Buffer was then added to give a final volume of 4 ml. per g. tumour tissue. The mash was left overnight at 4° C. The following day, it was centrifuged 3 times at high speed (10,000 × *g*), for 20 minutes each time. (Usually there still was a sediment after the second centrifugation, and the third was carried out to completely remove all sedimentable material. The third centrifugation could however be omitted if no sediment were obtained after the second centrifugation). The clear supernatant was distributed in ampoules and kept frozen. For electrophoretic and immunoelectrophoretic studies of tumour extracts, a sample containing a constant amount of protein was applied to the plates (i.e. 500–600 micrograms).

*Mouse sera*

Blood of normal and tumour-bearing mice was obtained by cardial puncture or puncture of the ophthalmic venous plexus with a capillary pipette. Tumour-bearing mice were bled 10–14 days after transplantation. Serum was separated from coagulated blood by centrifugation. Sera from 8–15 mice were pooled and stored frozen until used.

*Mouse urine*

Urine was obtained from male mice while stimulating them in the chest region with a single electric impulse (100 v) of short duration (<1 second); this treatment did not appear to cause any pain to the animals. Urine from tumour-bearing mice was collected 9–15 days after tumour transplantation. The urine was pooled, dialysed for 12–16 hours against phosphate-buffered saline (pH 7·4) and concentrated 4–5 times its original volume by perventilation. Volumes containing 500–600 μg. of protein were applied to agar plates for immunochemical examination.

*Antisera*

Antisera were produced in rabbits. Antisera to MMCIA and to urine of tumour-bearing mice were prepared as described previously (Pikovski and Witz, 1961*b*). Antisera to normal sera and urine of mice were prepared according to the immunization schedule described by de Vaux St. Cyr and Hermann (1963). These two immunization schedules were compared by immunizing rabbits with the same antigens (mouse sera and tumour extracts), in both ways, and then by analysing the precipitation patterns given by the resulting antisera. No significant differences in the patterns of the two antisera obtained by the two ways of immunization were detected. Also used in this study were antisera to the following fractions :

(1) Mouse gamma globulin and transferrin obtained by DEAE cellulose chromatography of mouse serum (Talal, Hermann, de Vaux St. Cyr and Grabar, 1963).

(2) Mouse transferrin and beta 2 III globulin. The preparation of this fraction is described further on in the present paper.

*Agar gel electrophoresis*

Agar gel electrophoresis was performed in 1 per cent agar gel, employing a barbital buffer pH 8·2, at a concentration of 0·025 M in the agar and 0·05 M in the reservoirs. The current was 6 v/cm. The plates measured 4·4 cm. by 10·7 cm. The duration of electrophoresis was 75 minutes.

*Immunochemical techniques*

Immunoelectrophoresis was performed according to Grabar and Williams (1953, 1955). The identification of the precipitation lines given by mouse serum constituents in the immunoelectrophoretic analyses was according to the nomenclature used by Heremans *et al.* (1959). In fresh sera the beta 3 I globulin shows a precipitation line with a single curve. If the serum is older than 24 hours changes occur, and this line becomes double curved. The other precipitation lines seem to remain unchanged. Proteins were stained with amido black after agar gel electrophoresis and with Ponceau S after immunoelectrophoresis, lipoproteins with sudan black and the esterases are visualized with beta-naphthylacetate and indoxylacetate as substrates with the methods indicated by Uriel (1960, 1961) and used previously (Talal, Hermann, de Vaux St. Cyr, 1962 ; Talal, Hermann, de Vaux St. Cyr and Grabar, 1963).

Haemoglobin binding was visualized with the method of Hirschfeld (1959), using human haemoglobin.

Antisera were absorbed by mixing 17–27 mg. of absorbing antigen with 1·0 ml. of antiserum. The mixture was incubated at 37° C. for 1 hour and then left at 4° C. over night. The following day it was centrifuged. Completion of absorption was checked by a negative reaction between absorbed antiserum and absorbing antigens.

*Protein estimation*

Proteins were determined with the Biuret reagent (Kabat and Mayer, 1961), and their concentration measured spectrophotometrically at 555 m$\mu$. Human serum albumin was used as a standard.

*Abbreviations*

Abbreviations used in this study were as follows :

I.E.A.—Immunoelectrophoretic analysis.
NMS—Normal mouse serum.
TMS—Serum from tumour-bearing mice.
TL—Extract from lyophilized tumour.
TF—Extract from fresh tumour.
NMU—Normal mouse urine.
TMU—Urine from tumour-bearing mice.

### RESULTS

### 1. *Tumour Extracts and Sera of Normal and Tumour-bearing Mice*

Agar gel electrophoresis of normal mouse serum and serum from tumour-bearing animals and tumour extracts was carried out parallel with immunoelectrophoretic analysis of these materials. The results obtained were as follows :

*Agar gel electrophoresis*

(1) Serum from tumour-bearing mice compared with NMS showed an increase in alpha globulins and a decrease in gamma globulins (Fig. 1A) and an increase in alpha lipoproteins (Fig. 1B). No difference in the esterase activity of the various protein fractions of the two types of sera was detected (Fig. 1C). The magnitude of these differences requires further work with other techniques and quantitative evaluation of the protein fractions.

(2) The tumour extracts showed the presence of at least three protein fractions migrating respectively in the region of serum albumin, alpha globulins and beta globulins (Fig. 1D). Lipoproteins were demonstrable with a migration somewhat slower than that found in normal serum (Fig. 1E). Analysis for esterases (Fig. 1F), showed the presence of esterase activity corresponding to Rho lipoprotein-esterase and the alpha 2 esterases of serum. Of particular interest however, is the presence of an esterase with an electrophoretic migration in the beta-gamma globulin region. An esterase of this type does not exist in serum. In all three zones a hydrolysis of beta naphtyl-acetate and of indoxyl-acetate occurred. No hydrolysis of acetyl-thiocholine or of butyryl-thiocholine was observed in any of the zones.

*Immunoelectrophoretic analyses (I.E.A.)*

Sera from normal and tumour-bearing mice were compared by I.E.A. Antisera to normal mouse serum (NMS) and to the mouse tumour extracts were used in this study. The antibody content of the two antisera differed with respect to certain specific proteins. The anti-NMS, for example, appeared to have a higher titre of antibodies against the beta 3 I globulin and a lower titre against the beta 2 I and the beta 2 III globulins than the anti-tumour serum. Comparisons between antigens was therefore made with the antiserum showing the highest titre for the protein to be examined. Criteria of increased concentration were an increase in density and length of a given precipitation line (Burtin, 1960).

The main difference between the proteins of NMS and TMS is found in an increased concentration of beta 2 I globulin and beta 2 III globulin which occur in the TMS (see Fig. 2A). This difference is much more obvious when the two sera are diluted. Fig. 2B shows the precipitation pattern obtained with NMS and TMS diluted 1 : 5. The diluted NMS almost did not produce the beta 2 III globulin arc (only a small fragment of the line is still visible), while the diluted TMS produced a strong line. The augmentation of the beta 2 I globulin in the TMS is less obvious, but nevertheless the diluted TMS produced a denser and longer beta 2 I globulin band which was also closer to the antibody trough than that produced by diluted NMS. All these facts indicate its higher concentration in the serum of tumour-bearing mice. In addition there is an indication of an increase of beta 3 I globulin.

I.E.A. of tumour extracts both from lyophilized (TL 1 and 2) and fresh tumour (TF) were also carried out against the two types of antisera mentioned above. The precipitation lines were more pronounced with antisera to tumour than with those to NMS (Fig. 3A). The beta 2 I and the beta 2 III lines were very conspicuous. The appearance of several lines, not produced by mouse serum, could be revealed by some immune sera to the tumour extracts. This was proved when analyses were carried out with these antisera absorbed with NMS. Three lines were evident when the absorbed sera reacted with the tumour extracts (Fig. 3B), revealing thus the presence in the tumour of non-serum antigens. I.E.A. revealed that these antigens had electrophoretic mobilities similar to beta and gamma globulins of serum.

The perfused fresh tumour extracts (TF) contained less of serum proteins than did the non-perfused lyophilized extracts (TL). This is possibly due to better removal of serum proteins in the first. The tissular non-serum proteins demonstrated in tumour extracts, could be better revealed in the lyophilized preparation.

It should be noted also that, although beta 3 I was an obvious constituent in TMS, it could not be demonstrated in tumour extracts. The area of the alpha globulins was too smeared (in relation to the separation normally obtained with mouse serum) to permit analysis.

### II. *Partial Purification of the Beta 2 I and Beta 2 III Proteins*

Because of the increased concentrations of the beta 2 I and beta 2 III globulins both in sera and in tumours, attempts were made to separate out these two protein fractions.

Since it has been suggested by Clausen and Heremans (1960) that the mouse beta 2 III is analogous to human beta 2 A, the fractionation procedure of Heremans, Heremans and Schultze (1959) for isolation of this protein was attempted on mouse sera and tumour fluids found in solid mammary tumours. The fluid is composed of serous, sometimes haemorrhagic exudate and interstitial fluid. It was found that both beta 2 globulins and other proteins (including albumin) were soluble in 0·1 M zinc sulphate. In order to eliminate these additional proteins, a preliminary separation with rivanol (diamino ethoxyacridine lactate) was carried out. The final fractionation procedure is shown in Fig. 4. The fraction thus obtained contains two proteins as shown in Fig. 5A ; they correspond in position to beta 2 I and beta 2 III found in both tumour extracts and in serum of MMCIA-bearing mice.

When antisera against this beta 2 globulin fraction were prepared, only two precipitation lines were obtained with whole mouse serum (Fig. 5B). This would indicate that the fractionation procedure resulted in only 2 proteins of different antigenic makeup.

Clausen and Heremans (1960) observed a cross-reaction between human transferrin and mouse beta 2 I globulin and thus concluded that this protein is the mouse transferrin. Definite proof for this assumption was presented by Clausen *et al.* (1960) using radioactive iron incorporation, and by Hermann and Bao-Dinh (1962, personal communication) by coloration of the iron bound to the transferrin, according to Uriel's method (unpublished data).

The haemoglobin binding capacity of mouse beta 2 III globulin was originally established by Velez and Hermann (1962, personal communication). These findings are confirmed in the present study. Haemoglobin was added to whole tumour extract and to the final globulin fraction, and immunoelectrophoresis carried out. The plate was photographed and then treated with benzidine in order to visualize the protein-bound haemoglobin by its peroxidase activity. The results of this test are shown in Fig. 6, which is a photomontage showing on the outer strips the pattern obtained after the immunoelectrophoretic procedure, and on the inner strips the benzidine-stained precipitation bands of the same plate. Aside from the spot corresponding to free haemoglobin, it can be seen that the beta 2 III lines of the fraction and of the tumour extract were stained, indicating that the protein has bound haemoglobin.

The identification of the second beta 2 globulin is shown in Fig. 7. A specific antiserum to mouse transferrin and gamma globulin was used in the I.E.A. The specificity of the antiserum is shown in the pattern obtained with NMS in which only two lines are evident (sometimes a third line—produced by beta

---

### EXPLANATION OF PLATES

FIG. 1.—Agar gel electrophoresis of mouse sera and tumour extracts.

A–C. Mouse sera (left of each pair—normal mouse serum (NMS)), (right of each pair—serum of tumour-bearing mice (TMS)).

D–F. Tumour extracts.

A, D are stained to visualize proteins.

B, E are stained to show lipoproteins.

C, F are stained to show esterase activity.

FIG. 2.—Immunoelectrophoretic analysis of mouse sera. NMS—normal mouse serum. TMS—serum from tumour-bearing mice. The arrow No. 1 indicates the precipitation arc given by beta 2 I globulin and No. III indicates beta 2 III globulin.

A. Note that (1) TMS, when reacted with anti-NMS produced a clear beta 3 I globulin line, while no beta 3 I globulin line was produced by NMS reacting with the same antiserum. (2) TMS gave longer and denser beta 2 III and beta 2 I lines than NMS. This is more evident in the following figure.

B. NMS and TMS diluted 1 : 5 were reacted with antiserum to tumour extracts. Note that in diluted NMS the beta 2 III line has become faint, and the beta 2 I line is shorter and less dense. On the other hand, diluted TMS had still sufficient amounts of these two beta globulins to produce conspicuous precipitation arcs. This plate also showed increase of some alpha globulins in the TMS, a finding which (although confirming the results of agar gel electrophoresis of TMS) appeared with only half of the antisera used.

FIG. 3.—A. Immunoelectrophoretic analysis of tumour extracts. $TL_1$ and $TL_2$—first and second extraction, respectively, of lyophilized tumour tissue. TF—extract of fresh tumour.

B. Ouchterlony plate, demonstrating absorption of antibodies to serum antigens from anti-tumour serum. Centre well : anti-tumour serum absorbed with mouse serum. Top and left wells : preparations of tumour extracts. Right well : normal mouse serum. Note that the tumour extracts produced at least three precipitation arcs with the absorbed antiserum. Mouse serum produced none.

FIGS. 5–7.—Purification and identification of beta 2 I and beta 2 III globulins.

Fig. 5.—A. Immunoelectrophoresis of the beta 2 globulin fraction as compared to serum and tumour extract, reacting with antiserum to tumour. B. Immunoelectrophoresis of serum and tumour extract reacting with the beta 2 globulin immune serum.

Fig. 6.—The indication of haemoglobin binding capacity of beta 2 III globulin. This figure is a photomontage showing the analysis of tumour extract and the beta 2 fraction with anti-tumour serum before and after benzidine staining. A–B. Tumour extract before (A) and after (B) the benzidine reaction. C–D. beta 2 globulin fraction before (D) and after (C) benzidine reaction. Note in both cases the presence of a benzidine positive line corresponding with the beta 2 III globulin line.

Fig. 7.—The identification of beta 2 I globulin as transferrin.

FIGS. 8–9.—Immunoelectrophoretic analysis of mouse urines.

Fig. 8.—Analysis of the constituents present in mouse urine as compared to serum. A. Utilization of antisera to urine. Photo of the plate. B. Schematic drawing of the immunoelectrophoretic patterns of normal mouse urine reaction with unabsorbed immune serum to urine (below) and absorbed with mouse serum (above). C. Reaction of mouse urine with immune serum to mouse serum. Note in this case that the only line appearing when urine reacted with antiserum to mouse serum, occurs faintly in the albumin region. It is not readily visible in the photographic reproduction.

FIG. 9.—A comparative analysis of antibodies present in antisera to urines of normal and tumour-bearing mice, using mouse sera as the antigens. Note that while antisera to normal mouse urine contains 2–3 precipitins against mouse sera, antisera to urine from tumour-bearing mice shows a number of additional precipitin lines, whose intensity is greater in the tumour serum.

Witz, Hermann, Pikovski and Gross.

Fig. 2

Fig. 3

Witz, Hermann, Pikovski and Gross.

Fig. 5

Fig. 6

Fig. 7

Witz, Hermann, Pikovski and Gross.

Fig. 8

Fig. 9

Witz, Hermann, Pikovski and Gross.

FIG. 4.—Scheme of the procedure for the purification of beta 2 I and beta 2 III globulins.

3 II globulin—is present). The reaction of this antiserum with the beta 2 globulin fraction resulted in the appearance of a single precipitation line corresponding to the transferrin line (beta 2 I) of NMS.

### III. *Urinary Proteins of Normal and Tumour-bearing Mice*

The urine contained a mixture of proteins, some of which were antigenically identical with those of mouse serum, and others were antigenically distinct from those of serum. This is illustrated in the following electrophoretic analyses.

When NMU was tested with the corresponding antiserum five to seven precipitation bands were evident (Fig. 8A, B). These may be described by their migration in relation to the equivalent migration of serum proteins. There is a large zone of protein extending from the prealbumin zone to the alpha 2 region (Rho-Alpha 2), albumin, three alpha 2 globulins, one precipitation line produced by a beta 1 globulin and one formed by a beta 2 globulin (Fig. 8A, B). When the urine antiserum was absorbed with normal mouse serum, two lines—those of albumin and of an alpha 2 globulin—disappeared. Five proteins, namely the Rho-Alpha 2, two alpha 2 globulins, one beta 1 and one beta 2 globulin were therefore indicated to be " urinary specific " proteins. Conversely, when urinary proteins were analysed with anti-NMS serum, an albumin line (Fig. 8C) and two lines with somewhat lesser migration (whose appearance was inconstant) were formed. Esterase activity was found in one of the alpha 2 lines and in agar gel electrophoresis also in the beta region. It would seem that the proteins in this region are in such low amounts or are of such low antigenicity that esterase activity is not demonstrable in a precipitation line.

Urines from tumour-bearing animals show a similar pattern of " urinary specific " protein distribution to that excreted by normal animals. However, there appeared to be increased amounts and types of antigens immunologically identical with serum proteins. When antisera against pathological urines reacted with serum proteins, the presence of antibodies against many protein constituents of serum was shown, whereas antisera to normal urine contained precipitating antibodies only against albumin and the alpha 2 globulin described above (Fig. 9).

### DISCUSSION

In the present study it was found that sera of cancerous animals contained higher than normal amounts of lipoproteins with alpha globulin mobility (in agar gel), haemoglobin binding beta 2 III globulin, beta 3 I globulin and transferrin. These results are in accordance with those obtained by other workers who dealt with sera of cancer patients and tumour-bearing animals (Clausen *et al.*, 1959, 1960 ; Rask-Nielsen, Gormsen and Clausen, 1959 ; Rabinovich de Piroski and Oisgold, 1962 ; Robert, Serpicelli and Jayle, 1956 ; Nyman, 1959 ; de Vaux St. Cyr, Cleve and Grabar, 1960). The significance of these findings in relation to the cancer process is still obscure.

The immunoelectrophoretic study of tumour extracts revealed that an esterase with a beta-gamma globulin mobility and some other proteins with similar mobility may be tissular components. However, the other proteins in tumour extracts, demonstrated by immunoelectrophoresis were either serum proteins or possibly tumour tissue proteins possessing common antigenic determinants with serum proteins.

The " urinary specific " antigens may represent either specific protein products of the kidney or the urinary tract (Boyce, King and Fielden, 1961) or, alternatively, split products of serum proteins as the result of metabolic activities of urinary tract cells. de Vaux St. Cyr, Hermann and Talal (1962, 1963) and Hermann (1963), working on human urine, have shown that it contained, in addition to various serum constituents and degraded gamma globulins, some fractions which were not demonstrable in serum, e.g. esterases and " uromucoide ".

The possibility that these types of protein may be secretions of the urinary tract has not as yet been excluded. The second possibility that split products with different antigenic sites may be obtained from one protein is supported by the work of Burtin (1961) on gamma globulin.

The difference in antigen patterns in urine from normal animals and from those bearing tumours is characterized by an increased number of serum antigens in the latter. These serum proteins could be demonstrated when the antiserum against TMU was tested against normal serum or serum of tumour-bearing animals. The latter showed more lines against anti-TMU than did normal serum.

These antigens could also be revealed by use of tumour extracts reacting with anti-TMU serum. When, however, this antiserum was absorbed with normal serum, no precipitation bands were formed. We could thus confirm our previous observation (Pikovski and Witz, 1961*b*) on the presence of certain tumour antigens in urine of tumour-bearing mice. These proteins are part of the group of tumour antigens possessing common antigenic sites with serum proteins, but might also be cell constituents. This point may be of special interest, bearing in mind the similarity in the effect on tumour cells produced by immune sera to tumour extracts and to urine of tumour-bearing mice (Pikovski, Witz and Tenenbaum, 1963).

The presence of serum proteins in TMU would suggest that if the kidney clearance of proteins into the urine remained constant, then in the tumour-bearing animal the increase of certain serum proteins (see above) would manifest itself in the amounts of these proteins present in the urine. However, an additional factor seems to be operating, since even those protein fractions that are not increased in the blood of tumour-bearing animals are present in the urine. This would indicate that perhaps in the tumour-bearing animal the kidney clearance of serum protein is somewhat elevated.

### SUMMARY

Growth of mammary carcinoma in mice was shown to be associated with increased concentrations of beta 2 I globulin (transferrin), beta 2 III globulin (a haemoglobin binding protein) and beta 3 I globulin (constituent of the complement) in serum.

Soluble extracts of tumour contained marked concentrations of the beta 2 I and the beta 2 III globulins. Three proteins with beta-gamma mobilities were detected and in addition esterase activity was demonstrable in the beta 2 region of the electropherogram. Neither the 3 latter proteins nor the esterase were demonstrable in serum.

The transferrin and the haemoglobin binding beta 2 III globulin were partially purified.

Normal mouse urine contained at least 5 to 7 proteins of which only two—albumin and an alpha globulin were identical with the corresponding serum proteins. Urine from tumour-bearing mice showed additional proteins, immunologically identical with those of serum and tumour extracts.

We are obliged to Professor P. Grabar and to Dr. C. de Vaux St. Cyr and Dr. P. Burtin for their constant interest, help, suggestions, and discussions of the work.

Part of the studies reported were carried out in the Institut de Recherches Scientifiques sur le Cancer, Villejuif, Seine, France, during the tenure of a stipend from the Anne Frank Foundation (Frankfurt/Main) by I. W., who is particularly grateful for the helpfulness of Professor P. Grabar, Director of the Institut de Recherches Scientifiques sur le Cancer, during his stay there.

This report is part of the Ph.D. work of I. W., holder of the Jacob I. Schaffer Scholarship.

The careful technical assistance of Miss I. Erk and Miss C. Brunschwig is acknowledged.

### REFERENCES

Boyce, W. H., King, J. S. and Fielden, M. L.—(1961) *J. clin. Invest.*, **40**, 1453.

Burtin, P.—(1960) in ' Analyse Immuno-Electrophoretique '. Edited by Grabar and Burtin. Paris (Masson and Cie), p. 81.—(1961) in ' Protides of the Biological Fluids '. Amsterdam (Elsevier Publishing Co.), p. 284.

CLAUSEN, J. AND HEREMANS, J.—(1960) *J. Immunol.*, **84**, 128.
*Idem*, HEREMANS, J., HEREMANS, M. T. AND RASK-NIELSEN, R.—(1959) *J. nat. Cancer Inst.*, **22**, 57.
*Idem*, RASK-NIELSEN, R., CHRISTENSEN, H. E. AND MUNKNER, T.—(1960) *Cancer Res.*, **20**, 178.
GRABAR, P., SELIGMANN, M. AND BERNARD, J.—(1955) *Ann. Inst. Pasteur*, **88**, 548.
*Idem* AND WILLIAMS, C. A.—(1953) *Biochim. biophys. Acta*, **10**, 193.—(1955) *Ibid.*, **17**, 67.
HEREMANS, J., CLAUSEN, J., HEREMANS, M. T. AND RASK-NIELSEN, R.—(1959) *J. nat. Cancer Inst.*, **22**, 45.
*Idem*, HEREMANS, M. T. AND SCHULTZE, H. E.—(1959) *Clin. Chim. Acta*, **4**, 96.
HERMANN, G.—(1963) *Verh. dtsch. Ges. inn. Med.*, 69. Kongress, Wiesbaden.
HIRSCHFELD, J.—(1959) *Acta path. microbiol. scand.*, **47**, 169.
KABAT, E. A. AND MAYER, M. M.—(1961) 'Experimental Immunochemistry', 2nd edition. Springfield, Illinois (Thomas, P.).
NYMAN, M.—(1959) *Scand. J. clin. Lab. Invest.*, **11**, Suppl. 39.
PIKOVSKI, M. A. AND WITZ, I. P.—(1961*a*) *Acta Un. int. Cancr.*, **17**, 244.—(1961*b*) *Brit. J. Cancer*, **15**, 584.
*Iidem* AND TENENBAUM, E.—(1963) *Nature, Lond.*, **198**, 1314.
RABINOVICH DE PIROSKY, R. AND OISGOLD, S.—(1962) *Rev. Soc. argent. Biol.*, **38**, 24.
RASK-NIELSEN, R., GORMSEN, H. AND CLAUSEN, J. A.—(1959) *J. nat. Cancer Inst.*, **22**, 509.
ROBERT, L., SERPICELLI, J. AND JAYLE, M. F.—(1956) *Rev. franc. Etud. clin. biol.*, **1**, 976.
TALAL, N., HERMANN, G. AND DE VAUX ST. CYR, CH.—(1962) in 'Protides of the Biological Fluids'. Amsterdam (Elsevier Publishing Company), p. 183.
*Iidem* AND GRABAR, P.—(1963) *J. Immunol.*, **90**, 246.
URIEL, I.—(1960) in 'Analyse Immuno-Electrophoretique', edited by Grabar and Burtin. Paris (Masson and Cie), p. 33.—(1961) *Ann. Inst. Pasteur*, **101**, 104.
DE VAUX ST. CYR, C., CLEVE, H. AND GRABAR, P.—(1960) *Rev. franc. Etud. clin. biol.*, **5**, 776.
*Idem* AND HERMANN, G.—(1963) *Ibid.* (In press).
*Idem*, HERMANN, G. AND TALAL, N.—(1962) *Verh. dtsch. Ges. inn. Med.*, 68. Kongress, Wiesbaden, 535.—(1963) *Rev. franc. Etud. clin. biol.*, **8**, 241.